# Supplementary material for: Male germ cell-associated kinase is required for axoneme formation during ciliogenesis in zebrafish photoreceptors
Source: Dis Model Mech. 2024 Jul 16;17(7):dmm050618. doi: 10.1242/dmm.050618 (PMC11273301; doi:10.1242/dmm.050618)
Supplement: Supplementary information [file dmm-17-050618-s1.pdf]

```
                   1                                                                              80
Consensus          MNRYTTMKQLGDGTYGSVLMGKSNESGELVAIKRMKRKFYSWDECMNLREVKSLKKLNHANVIKLKEVIRENDHLYFVFE

zMAK               MNRYTTLKQLGDGTYGSVLMGKSNESGELVAIKRMKRKFYSWEECMNLREVKSLKKLNHANVVKLKEVIRENDHLYFVFE
mMAK               MNRYTTMKQLGDGTYGSVLMGKSNESGELVAIKRMKRKFYSWDECMNLREVKSLKKLNHANVIKLKEVIRENDHLYFVFE
hMAK               MNRYTTMRQLGDGTYGSVLMGKSNESGELVAIKRMKRKFYSWDECMNLREVKSLKKLNHANVIKLKEVIRENDHLYFIFE

                   81                                                                            160
Consensus          YMKENLYQLMKDRENKLFPESVIRNIMYQILQGLAFIHKHGFFHRDMKPENLLCMGPELVKIADFGLARELRSQPPYTDY

zMAK               YMKENLYQLMKDRENKMFTENEIRNIMFQVLSGLAFVHKHGFFHRDMKPENLLCMGPELVKIADFGLAREIRSRPPYTDY
mMAK               YMKENLYQLMKDR-NKLFPESVIRNIMYQILQGLAFIHKHGFFHRDMKPENLLCMGPELVKIADFGLARELRSQPPYTDY
hMAK               YMKENLYQLMKDR-NKLFPESVIRNIMYQILQGLAFIHKHGFFHRDMKPENLLCMGPELVKIADFGLARELRSQPPYTDY

                   161                                                                           240
Consensus          VSTRWYRAPEVLLRSSVYSSPIDVWAVGSIMAELYTLRPLFPGTSEVDEIFKICQVLGTPKKSDWPEGYQLASSMNFRFP

zMAK               VSTRWYRAPEVLLRSPVYSSPIDIWAVGCIMAELYTLRPLFPGNSEVDEIFKICQVLGTVKKSDWPEGHQLASAMNFRFP
mMAK               VSTRWYRAPEVLLRSSVYSSPIDVWAVGSIMAELYTFRPLFPGTSEVDEIFKICQVLGTPKKSDWPEGYQLASSMNFRFP
hMAK               VSTRWYRAPEVLLRSSVYSSPIDVWAVGSIMAELYMLRPLFPGTSEVDEIFKICQVLGTPKKSDWPEGYQLASSMNFRFP

                   241                                                                           320
Consensus          QCVPINLKTLIPNASNEAIQLMTEMLNWDPKKRPTASQALKHPYFQVGQVLGPSAHHLDSKQALHKQLQPLELKPSLQEV

zMAK               QCVPTPLKTLIPNATNEALDIMRDLLQWDPKKRPSAVKALRYPYFQVGQLLGPRPMTPDVRKAPVKAVLPSELRSEMQSV
mMAK               QCIPINLKTLIPNASSEAIQLMTEMLNWDPKKRPTASQALKHPYFQVGQVLGSSAHHLDTKQTLHKQLQPLEPKPSSSER
hMAK               QCVPINLKTLIPNASNEAIQLMTEMLNWDPKKRPTASQALKHPYFQVGQVLGPSSNHLESKQSLNKQLQPLESKPSLVEV

                   321                                                                           400
Consensus          DPKPLPDIIDQPAGQPQPKNSHQPLQPIQPPQNLSQHDPPKQQGHEKPPQTLFPSIIKKIPGGENSTLGHKGGRRRWGQT

zMAK               SE---PVLPSQ--TKGQSRNSHQPLQQIPLPQSLTQTDTVA-------------LHTQKVVGGENSLMGLKNGRRRWGQT
mMAK               DPKPLPNILDQPAGQPQPKQGHQPLQTIQPPQNTVTHPPPKQQGHQKPPQTMFPSIIKTIPVNSVSTLGHKGARRRWGQT
hMAK               EPKPLPDIIDQVVGQPQPKTSQQPLQPIQPPQNLSVQQPPKQQSQEKPPQTLFPSIVKNMPTKPNGTLSHKSGRRRWGQT

                   401                                                                           480
Consensus          IFKSGDSWDDIEDSDFGASHSKKPSMGACKEKRKKDSPFRFPDPGFSGSNHFKGENKKLPATSRSLKSDSELSTASTAKQ

zMAK               NMKPSDSWDDSDCSDTAASLSKKPSKGPEEEKTTPQPKEQKPLYTFST-------VTKLPNTSRLNQSDSSHQSTSSARQ
mMAK               VFKSGDSCDDIED-DLGASHSKKPSMEACKEK-KKESPFRFPDSGLPVSNHFKGENRNLHA-SVSLKSDPNLSTASTAKQ
hMAK               IFKSGDSWEELEDYDFGASHSKKPSMGVFKEKRKKDSPFRLPEPVPSGSNHSTGENKSLPA-VTSLKSDSELSTAPTSKQ

                   481                                                                           560
Consensus          YYLKQSRYLPGVNPKNVSLIASGKEINPHFWDNSQLFPKSLGPIGAALAFKRSNAEDSIIKPIEKLSCNEKFAEKLEDPQ

zMAK               HYLRQSRYMPGLISKNTQSLSSK-ESQQDFWDTSALMSKPLAPIGGAPMS-------------------------RISP
mMAK               YYLKQSRYLPGVNPKNVSLVAGGKDINSHSWNN-QLFPKSLGSMGADLSFKRSNAEDSIIKPIENLSCTGKSAEQLEDPQ
hMAK               YYLKQSRYLPGVNPKKVSLIASGKEINPHTWSN-QLFPKSLGPVGAELAFKRSNAEESIIKPIEKLSCNETFPEKLEDPQ

                   561                                                                           640
Consensus          GNLGSYTTYNQGGYIPSFLKKEVGSAGQRIQLAPLGAQASLDLSATADCKTGKAKPSKSKPSSTVSVNDNSEDDYTWKTK

zMAK               GNFVTTTYNLSGGYIPSFQKKEVGSVGQRIQLAPLGHQHALDLSATADCKTGKAKPSKSKPSSTVSVNDNSEDYDGWKRR
mMAK               GNLGSYTTYNQTGYMPSFLKKEVGSAGQRIQLAPLGASAS---------------------------------DYTWSTK
hMAK               GNLGSYATYNQSGYIPSFLKKEVQSAGQRIHLAPLNATAS---------------------------------EYTWNTK

                   641                                     685
Consensus          TGRGQFSGRTYNPTAKNSLNIVNRAQPVPSVHGRTDWVAKYGGHR

zMAK               AERTQLKGTSYSALGKNSANLITHAPSVQPVHGRIDWAAKYGSHR
mMAK               TGRGQFSGRTYNPTAKN-LNIVNRTQPVPSVHGRTDWVAKYGGHR
hMAK               TGRGQFSGRTYNPTAKN-LNIVNRAQPIPSVHGRTDWVAKYGGHR
```

Amino acid sequence identity percentage

	Full length	Kinase domain	C'-terminus
zMAK vs mMAK	50% (323/646)	86.17% (243/282)	21.82% (79/362)
zMAK vs hMAK	51.38% (333/648)	88.65% (250/282)	21.97% (80/364)
mMAK vs hMAK	83.64% (542/648)	97.86% (275/281)	72.52% (264/364)
Among the three animals	47.22% (306/648)	85.10% (240/282)	17.03% (62/364)

Fig. S1. Amino acid comparison between zebrafish, mouse, and human MAK.

Amino acid sequences of zebrafish, mouse, and human MAK proteins are aligned using Clustal Omega. Grey shading indicates sequence differences. The kinase domain is indicated by an underline. The bottom table shows the percentage of amino acid identity in the full-length protein, the kinase domain, and the C-terminal domain.

Disease Models & Mechanisms • Supplementary information

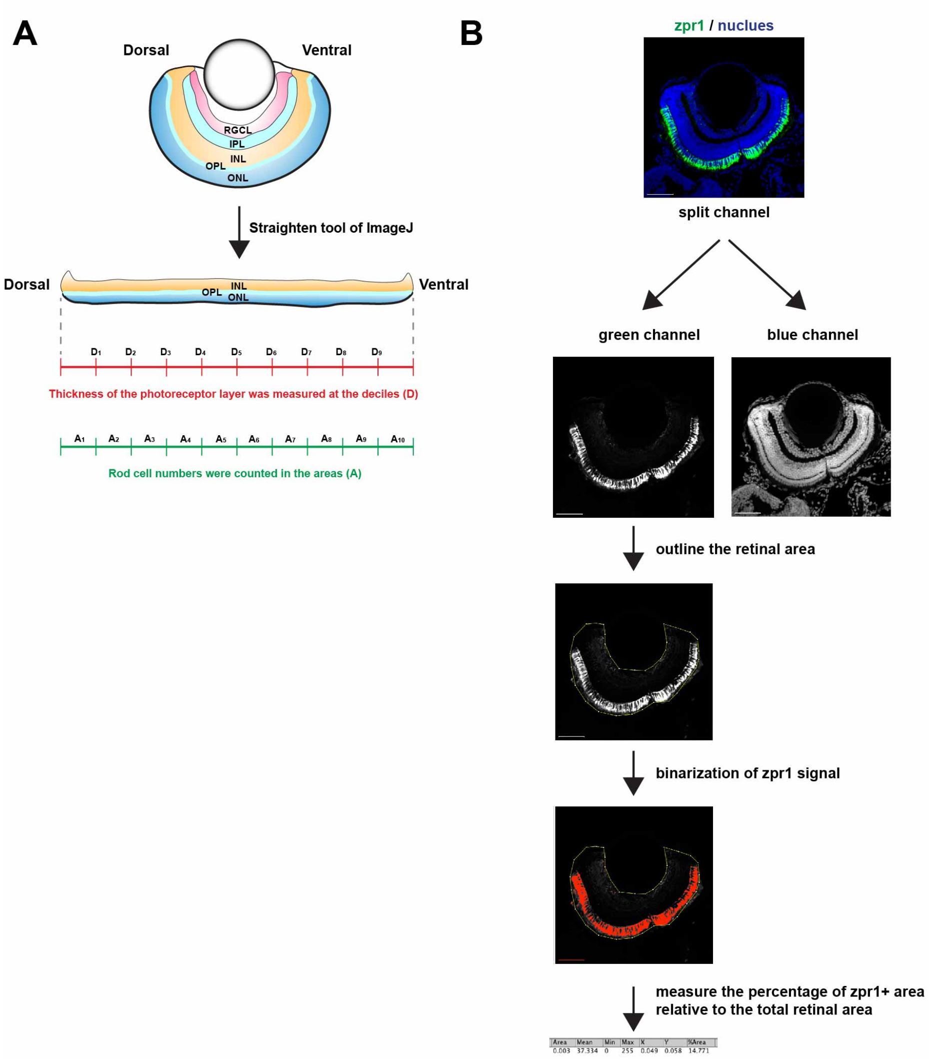

Fig. S2. Measurement of ONL thickness, rod cell number, and the fraction of zpr1-positive area with regard to total retinal area.

(A) Images with retinas were straightened with Image J (Fiji). Straightened images were divided into 10 equal areas (A) with 9 deciles (D) between adjacent areas. The thickness of the ONL was measured at the deciles and cell numbers were counted in each area.

(B) Cryosections labeled with zpr1 antibody were scanned under a laser-scanning microscope (LSM510; Carl Zeiss, FV3000; Olympus). First, a one-section image containing the central retina per eye was selected from each individual sample. Using the "split channel" command of Image J (Fiji), confocal scanning RGB color images were split into either R, G or B channel. Only the zpr1 channel (green channel in this figure) is used to draw the outline of the total retinal area. Next, using the "threshold" command of Image J (Fiji), we converted zpr1-positive and -negative areas to a binary scale, 1 and 0, respectively. The number of pixels corresponding to 1 or 0 within the neural retina was determined. Using the "measurement" command of Image J (Fiji), the percentage of zpr1-positive areas relative to the total retinal area was calculated as the percentage of the number of 1 pixels relative to the number of 0+1 pixels. Means and standard deviations were calculated from data obtained for 2-5 sections of more than two embryos.

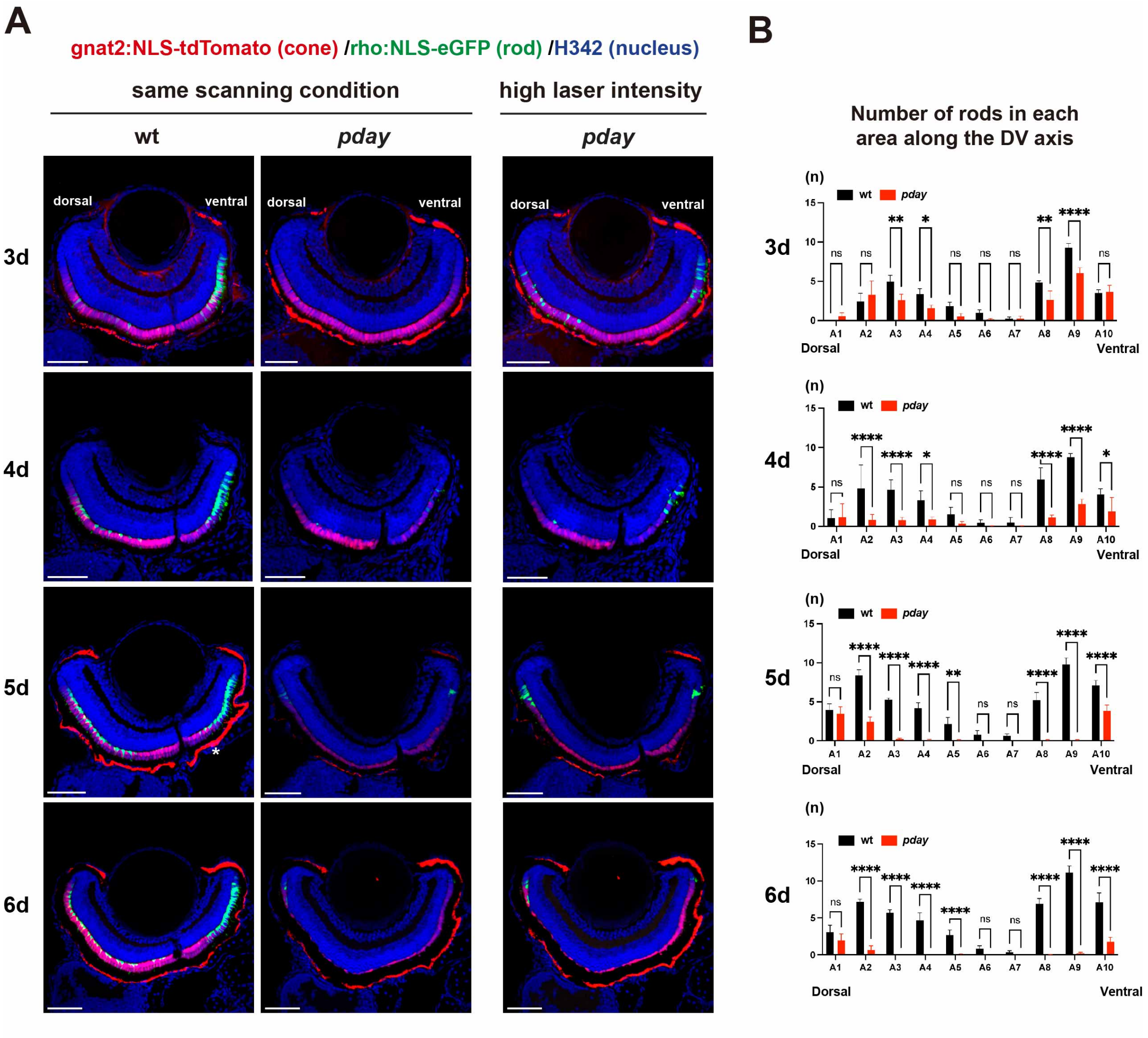

Fig. S3. Visualization of rods and cones in *pday* mutants.

(A) Confocal images of wild-type (the left column) and *pday* mutant retinas (middle column) carrying the transgenes *Tg[rho:NLS-eGFP]* and *Tg[gnat2:NLS-tdTomato]*, which visualize rods (green) and cones (red), respectively. Nuclei were counterstained with Hoechst 33342 (blue). In *pday* mutant retinas, eGFP signals were weak. The right column indicates confocal scanning images of *pday* mutant retinas carrying the transgenes *Tg[rho:NLS-eGFP]* and *Tg[gnat2:NLS-tdTomato]*, with higher laser intensity, to enhance eGFP signals. We compare wild-type and *pday* mutant retinas in conventional scanning conditions and with higher laser intensity, respectively, in Figure 2. Scale bars: 40 μm.

(B) Histogram of rod numbers of wild-type siblings (black) and *pday* mutants (red) retinas at 3, 4, 5, and 6 dpf. Rod number was counted in 1/10th divided area of the ONL along the DV axis and analyzed with 2-way ANOVA and Sidak's multiple comparison tests, n=3 for wild-type, n=4 for *pday* mutants at 3 dpf; n=4 for wild-type, n=5 for *pday* mutants at 4 dpf; n=3 for wild-type, n=3 for *pday* mutants at 5 dpf; n=4 for wild-type, n=4 for *pday* mutants at 6 dpf. Bars and lines indicate mean ± SD. * p<0.02, ** p<0.003, **** p<0.0001, ns: not significant.

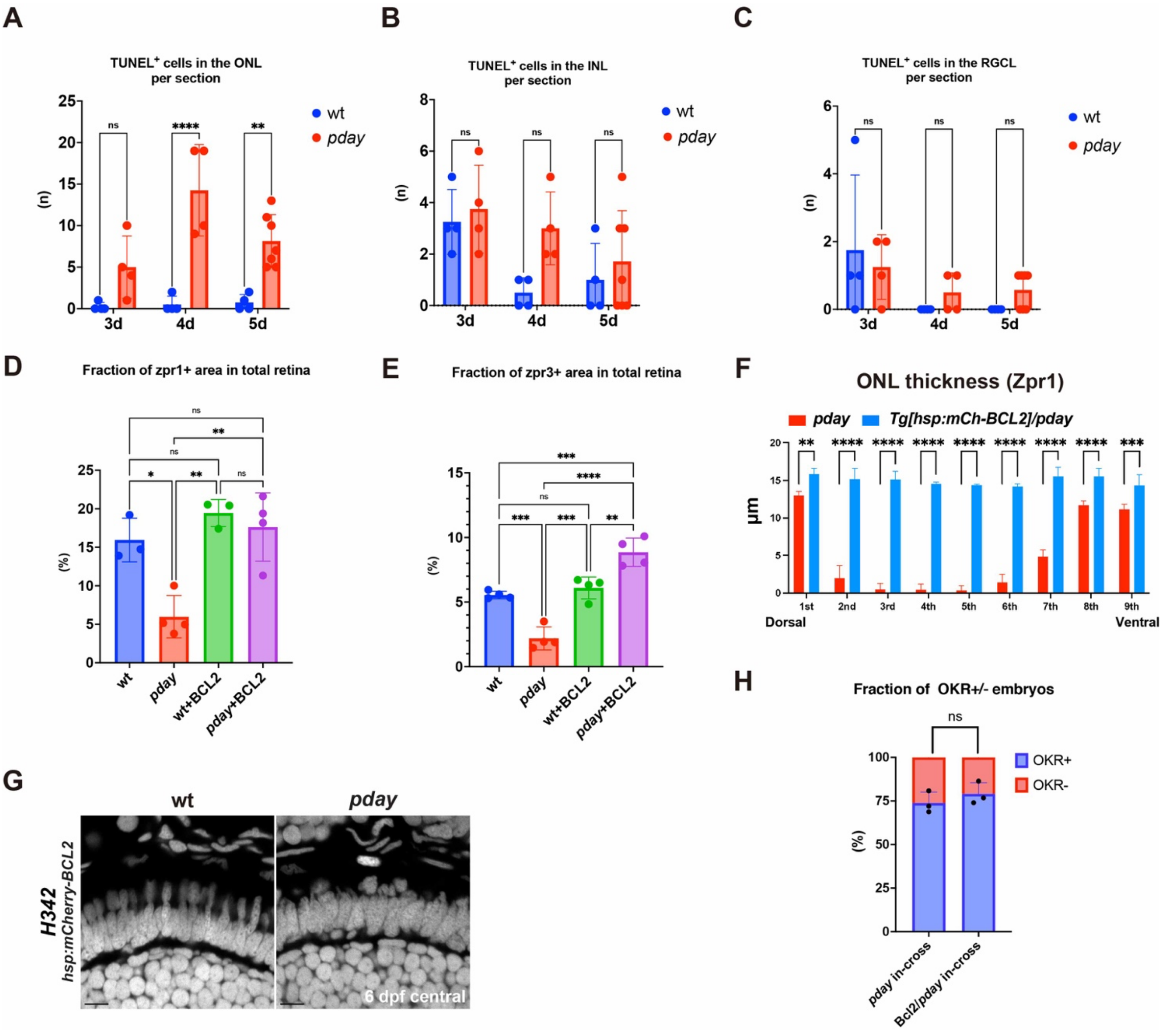

Fig. S4. Statistical analysis of TUNEL and Bcl2-mediated rescue of photoreceptor phenotypes in *pday* mutants.

(A) The number of TUNEL signals in wild-type and *pday* mutant ONL at 3, 4, and 5 dpf. TUNEL signals in the ONL are higher in *pday* mutants than in wild type at 3 dpf, although the difference is not significant. TUNEL signals in the ONL are the highest in *pday* mutants at 4 dpf and are still significantly higher in *pday* mutants at 5 dpf, compared with wild-type siblings. Bars and lines indicate mean ± SD. Statistical significance was evaluated with 2-way ANOVA and Sidak's multiple comparison tests; * p<0.03, **** p<0.0001, ns: not significant.

(B) The number of TUNEL signals in wild-type and *pday* mutant INL at 3, 4, and 5 dpf. TUNEL signals in the INL are higher in *pday* mutants than in wild type at 4 dpf, although the difference is not significant. Bars and lines indicate mean ± SD. Statistical significance was evaluated with 2-way ANOVA and Sidak's multiple comparison tests; ns: not significant.

(C) The number of TUNEL signals in wild-type and *pday* mutant RGCL at 3, 4, and 5 dpf. There is no significant difference between wild-type and *pday* mutants at any stage. Bars and lines indicate

mean ± SD. Statistical significance was evaluated with 2-way ANOVA and Sidak's multiple comparison tests; ns: not significant.

(D) The fraction of zpr1-positive area in the total retinal in wild-type and *pday* mutants without and with mCherry-Bcl2 overexpression. The fraction of the zpr1-positive area is markedly reduced in *pday* mutants (red bar), compared with wild-type siblings (blue bar). However, the fraction of the zpr1-positive area is recovered in *pday* mutants with mCherry-Bcl2 overexpression (purple bar), equivalent to wild-type without (blue bar) and with mCherry-Bcl2 overexpression (green bar). Bars and lines indicate mean ± SD. Statistical significance was evaluated with 2-way ANOVA and Turkey's multiple comparison tests; *p<0.0332, **p<0.0021, ns: not significant.

(E) The fraction of the zpr3-positive area in the total retinal area in wild-type and *pday* mutants without and with mCherry-Bcl2 overexpression. The fraction of the zpr3-positive area is markedly reduced in *pday* mutants (red bar), compared with wild-type siblings (blue bar). However, the fraction of the zpr3-positive area is recovered and increased in *pday* mutants with mCherry-Bcl2 overexpression (purple bar), compared with wild-type without (blue bar) and with mCherry-Bcl2 overexpression (green bar), probably due to ectopic distribution of zpr3 signals outside the OS. Bars and lines indicate mean ± SD. Statistical significance was evaluated with 2-way ANOVA and Turkey's multiple comparison tests; **p<0.0021, ***p< 0.0002, ****p<0.0001, ns: not significant.

(F) Histogram of zpr1-positive cone thickness in *pday* mutant retinas with (blue) and without (red) overexpression of mCherry-Bcl2. The ONL is divided into 10 areas along the DV axis. zpr1-positive cone thickness was measured at the interface between each neighboring region and evaluated with 2-way ANOVA and Sidak's multiple comparison tests, n=3 for *pday* mutants, n=4 for *pday* mutants with *Tg[hsp:mCherry-Bcl2]*. ** p<0.003, *** p<0.001, **** p<0.0001. Bars and lines indicate mean ± SD.

(G) Nuclear labeling of wild-type and *pday* mutant ONL overexpressing mCherry-Bcl2 with Hoechst 33342. Nuclear shape in the ONL is slightly irregular in *pday* mutants overexpressing Bcl2.

(H) The percentage of OKR+ (blue) and OKR- (red) embryos produced by crosses of *pday* heterozygous male and female fish without and with the transgene *Tg[hsp:mCherry-Bcl2]*. There is no significant difference between the absence and the presence of *Tg[hsp:mCherry-Bcl2]*. Statistical difference as evaluated with 2-way ANOVA and Sidak's multiple comparison tests. Bars and lines indicate mean ± SD, ns: not significant.

Scale bars: 5 μm (G).

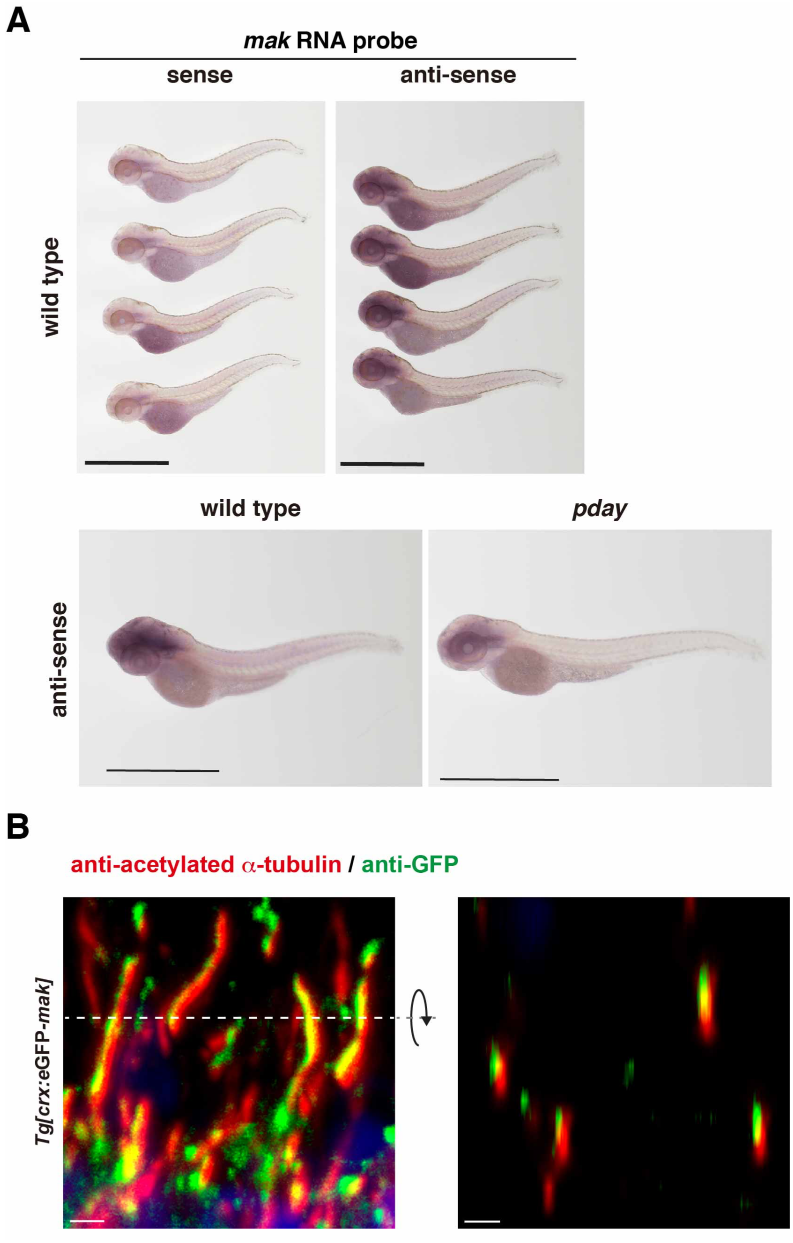

Fig. S5. *mak* mRNA expression is reduced in *pday* mutants and MAK protein is localized in ciliary axonemes.

(A) *In situ* hybridization of zebrafish embryos at 72 hpf with *mak* RNA probe. Upper panels indicate *in situ* hybridization with sense (left) and anti-sense RNA probes (right). Bottom panels indicate *in situ* hybridization of wild-type and *pday* mutant embryos with *mak* anti-sense probe. In pday mutant embryos, *mak* mRNA expression is reduced. Scale bars: 1 mm.

(B) Projection view of 3D confocal image of wild-type transgenic *Tg[crx: eGFP-MAK]* ONL with anti-GFP and anti-acetylated α-tubulin antibodies. Nuclei were counterstained with Hoechst 33342. Left panel indicates a radially sectioned projection view of the retina which is shown in the right panel of Fig. 5C. The eGFP-MAK signals overlap with anti-acetylated α-tubulin signals. Right panel indicates tangentially sectioned projection view, which was digitally produced by rotating the radially sectioned view using Imaris software (Bitplane). Scale bars: 1 μm.

Disease Models & Mechanisms • Supplementary information

Table S1. List of primers

NAME	SEQUENCE (5' TO 3')	REMARK
z23011_Fw	TTGGGGAAAAAGTCTGCTTG	SSLP BSA
z23011_Rev	TCCAACATGTAAAAACCTGACAA	SSLP BSA
mak-N1_Fw	GTGCAAGGTGTGAATTAGAC	SSLP BSA
mak-N1_Rev	AACTTCCCGACTTCTGTAAC	SSLP BSA
mak_Fw3	ACAGTGGGATGGAGTGGCATCCCTT	mak cDNA cloning
mak_Rev3	TTTCCGAGCGTTGAAAGTAGTCC	mak cDNA cloning
mak-I4_Fw	TGATGTTCACCAGCCACACT	pday site sequence
mak-I3_Rev	TCCCAGTTTTGATGTTGCAT	pday site sequence
mak-E5_Fw	CACAAATGCCAAACCAGAGA	pday site sequence
mak-E4_Rev	TGGTGAAGCTAAAGGAGGTGA	pday site sequence
MAK-AR_Fw	AGTGGCCATCgcgAGAATGAAGAG	AR MAK mutagenesis
MAK-AR_Rev	CTCTTCATTCTcgcGATGGCCACT	AR MAK mutagenesis
MAK-ADF_Fw	cttcGTCTCAACACGATGGTATCGAG	ADF MAK mutagenesis
MAK-ADF_Rev	tctgcGTAAGGTGGCCGAGAGCG	ADF MAK mutagenesis
arl13b_Fw	GGAAATGTTCAATCTGATGGCGAACTG	arl13b cDNA cloning
arl13b_Rev	GTGTTAGGAAATGACATCATGAGTATCGC	arl13b cDNA cloning
T3_mak_Fw	CTAATTAACCCTCACTAAAGGATGAACCGTTACACGACACTCAAG	mak in situ probe
mak_T7_Rev	CTTAATACGACTCACTATAGGGTTAACGATGGCTTCCATATT	mak in situ probe
Z13695_Fw	TGTCGTAGGACATGGGGATT	BSA SSLP
Z13695_Rev	TCGCTGCTTCGTTCTCACTA	BSA SSLP

Disease Models & Mechanisms • Supplementary information

Table S2. List of antibodies

NAME	SPECIES	SOURCE	USAGE	REMARK
Zpr-1	Mouse mAb	ZIRC	IHC: 1:100	
Zpr-3	Mouse mAb	ZIRC	IHC: 1:100	recognize zebrafish green opsin and rhodopsin (Gao et al., 2022)
Anti-Rhodopsin	Mouse mAb, clone 1D4	Abcam, ab5417	IHC: 1:100	recognize zebrafish red opsin (Yin et al., 2012)
Anti-GFP	Rabbit pAb	Invitrogen, A11122	IHC: 1:100	
Anti-acetylated Tubulin	Mouse mAb, clone 6-11B-1	Sigma Aldrich, T7451	IHC: 1:250	
Anti-EYS	Rabbit pAb	Novus Bio, NBP1-90038	IHC: 1:300	pH 6.0 HIAR
Anti-gamma Tubulin	Mouse mAb, clone GTU88	Sigma Aldrich, T6557	IHC: 1:100	pH 6.0 HIAR
Anti-MAK	Rabbit pAb	Scrum Inc.	WB: 1:1000	custom ab
Anti-MBP	Mouse mAb, clone SKB3	Merck, 05-675	WB: 1:5000	
Anti-phospho-MBP	Mouse mAb, clone P12	Merck, 05-429	WB: 1:10000	
Anti-GST	Mouse mAb, clone B-14	Santa Cruz, sc-138	WB: 1:10000	
Anti-zebrafish rhodopsin	Rabbit pAb	ZFIN: ZDB-ATB-09058-5	IHC: 1:5000	Ref. Vihtelic and Hyde, 1999 (DOI: 10.1017/s0952523899163168)
Anti-CEP290	Rabbit pAb	Abcam, ab84870	IHC: 1:100	

Table S3. Experimental sample sizes

Available for download at
https://journals.biologists.com/dmm/article-lookup/doi/10.1242/dmm.050618#supplementary-data
